# The Role of MicroRNA in DNA Damage Response

**DOI:** 10.3389/fgene.2022.850038

**Published:** 2022-05-03

**Authors:** Yongxin Li, Yan Tong, Jiaqi Liu, Jianlin Lou

**Affiliations:** ^1^ School of Public Health (Institute of Occupational Diseases), Hangzhou Medical College (Zhejiang Academy of Medical Sciences), Hangzhou, China; ^2^ Affiliated Hangzhou First People’s Hospital Zhejiang University School of Medicine, Hangzhou, China

**Keywords:** microRNA, DNA damage response, cell cycle, DNA damage repair, apoptosis

## Abstract

DNA is essential for the development and function of organisms. A number of factors affect DNA integrity and cause DNA damages, such as ultraviolet light, ionizing radiation and hydrogen peroxide. DNA damages activate a series of intracellular reactions, called DNA damage response, which play a crucial role in the pathogenesis of cancers and other diseases. MiRNA is a type of evolutionarily conserved non-coding RNA and affects the expression of target genes by post-transcriptional regulation. Increasing evidences suggested that the expression of some miRNAs was changed in tumor cases. MiRNAs may participate in DNA damage response and affect genomic stability via influencing the processes of cell cycle, DNA damage repair and apoptosis, thus ultimately impact on tumorigenesis. Therefore, the role of miRNA in DNA damage response is reviewed, to provide a theoretical basis for the mechanism of miRNAs’ effects on DNA damage response and for the research of new therapies for diseases.

## 1 Introduction

Since microRNAs (miRNAs) were first discovered by Lee *et al.* in 1993 to regulate embryonic development of nematode worms, more and more studies have found that miRNAs are prevalent in mammals (Lee et al., 1993). A mature miRNA is single-stranded non-coding RNA and evolutionarily conserved (Majidinia and Yousefi, 2016). It is commonly believed that miRNAs could directly regulate the abundance of a variety of mRNAs, hence participate in cell growth, aging, apoptosis and other life activities, affecting the occurrence and progression of multiple diseases (Wijnhoven et al., 2007).

Endogenous and exogenous stimuli may induce DNA damages and affect DNA replication and transcription processes, such as ultraviolet radiation, ionizing radiation, oxidants, chemotherapy and radiotherapy drugs. Excessive accumulation of DNA damages is directly related to the stability and integrity of the genome and endangers individual health (Majidinia and Yousefi, 2016; Ou and Schumacher, 2018). Recent findings have verified that miRNAs regulate three important aspects of DNA damage response (DDR)-cell cycle, DNA damage repair and apoptosis, forming a complex regulatory network (Majidinia and Yousefi, 2016). The levels of miR-29b, miRNA-320c and miR-1258 were decreased respectively in prostate cancer, glioma and colorectal cancer, and overexpression of the three miRNAs significantly affected DDR by inducing apoptosis or blocking cell cycle (Lv et al., 2018; Zhang et al., 2018; Sur et al., 2019), revealed that miRNAs might play a key role in life activities mediated by DDR. In this paper, the specific role of miRNA in DDR is reviewed to fully understand the general pattern of miRNA in DDR regulation.

## 2 Overview of MiRNAs

MiRNAs are evolutionarily conserved small single-stranded RNA molecules encoded by endogenous genes (Majidinia and Yousefi, 2016). The length of miRNAs are about 18–25 nucleotides (Majidinia et al., 2020). MiRNAs are not translated into proteins, but influence a variety of biological processes by post-transcriptional regulation (McManus, 2003; Majidinia et al., 2020). MiRNAs can reduce the expression of proteins via degrading the target mRNA or suppressing target mRNA translation by complete or incomplete complementary base pairing (Majidinia and Yousefi, 2016). One miRNA can regulate the expression of multiple target genes, whereas several miRNAs can work together on one target gene to regulate its expression as well, forming an intricate regulatory network. In order to distinguish the same miRNA among different species, the name of a miRNA can be preceded by a Latin abbreviation of species name (Lai et al., 2021). For example, miR-762 in human and mouse was expressed by hsa-miR-762 and mmu-miR-762 respectively (Lai et al., 2021).

Biosynthesis of miRNAs require transcription, processing, maturation and degeneration (Majidinia and Yousefi, 2016). In mammalian cells, miRNA synthesis is typical initiated by the transcription of miRNA genes, which is dependent on RNA polymerase II in the nucleus and promotes the generation of primary miRNAs (Majidinia and Yousefi, 2016). Then primary miRNAs are processed into about 70 nucleotides in the nucleus by Drosha-DiGeorge syndrome chromosomal region 8 complex, named precursor miRNAs (Michlewski and Caceres, 2019). With the help of nuclear transporter exprotin-5, the hairpin structured precursor miRNAs are transported out of the nucleus and spliced into double-stranded miRNAs by endoribonuclease Dicer in the cytoplasm (Michlewski and Caceres, 2019; Majidinia et al., 2020). One of the miRNA strands is mature and then would be integrated into the RNA-induced silencing complex together with an argonaute protein, which results in mRNAs degradation or repression of target mRNAs translation (Michlewski and Caceres, 2019). Finally, the mature miRNAs are degraded after completing its biological function (Michlewski and Caceres, 2019).

## 3 DNA Damage Response

Endogenous and exogenous stimuli lead to genomic DNA instability and DNA damages, such as ionizing radiation, chemotherapeutic agents, ultraviolet radiation and reactive oxygen species (Ou and Schumacher, 2018). At this point, the complex cascade reactions known as DDR is activated in eukaryotic cells (Jackson and Bartek, 2009). Firstly, the DNA damage is recognized by intracellular sensors such as Mre11/Rad50/NSB1(MRN) complex (Fang and Pan, 2019). Subsequently the phosphatidylinositol-3-kinase (PI3K)-related kinase including ataxia telangiectasia mutated (ATM), ATM- and Rad-3-related protein (ATR) and DNA-PKcs are phosphorylated as mediators (Yue et al., 2020). Usually, ATR is typically activated in DNA single-strand breaks (SSBs), while ATM and DNA-PKcs are typically activated in DNA double-strand breaks (DSBs) (Yue et al., 2020). For instance, ATM is recruited to DNA damage sites by MRN complex. Then the histone H2AX is rapidly phosphorylated, which is an attractive biomarker of DDR (Kopp et al., 2019). Then the signals are transmitted to downstream effectors via a cascade reaction mediated by signal molecules, so as to activate the cell cycle checkpoints correctly and induce cell cycle arrest (Jackson and Bartek, 2009). Members of cyclin-dependent kinases (CDKs) family-CDK1, CDK2, CDK4 and CDK6- play a pivotal role in regulating cell cycle transition and be affected by CDK inhibitor 1A (p21), CDK inhibitor 1B (p27), CDK inhibitor 2A and so on (Haferlach et al., 2011; Chou et al., 2020).

Concurrent with the cell cycle adjustment, the DNA repair pathways are activated to repair the damaged DNA. The ways of DNA damage repair mainly include base excision repair (BER), nucleotide excision repair (NER), homologous recombination repair (HR) and non-homologous end joining repair (NHEJ) (Mota et al., 2019). DNA double-strand breaks (DSBs) lead to severe consequences and can be repaired by HR and NHEJ (Piotto et al., 2018). If the DNA damages cannot be repaired, apoptosis is activated to remove the impaired cells (Ou and Schumacher, 2018). Apoptosis is a kind of orderly pathway of cell death that maintains homeostasis and destroys damaged or unwanted cells in eukaryotes (Singh et al., 2019). The internal or external stimuli induces apoptosis, and activated two different pathways: the mitochondria-mediated intrinsic pathway and the death receptor-mediated extrinsic pathway (Singh et al., 2019). The B cell lymphoma 2 (BCL-2) family and Caspase family play key roles in apoptosis (Singh et al., 2019; Van Opdenbosch and Lamkanfi, 2019). And BCL-2 family consists of pro-apoptotic factor BCL-2-Associated X (BAX), BCL-2-interacting mediator of cell death (BIM), as well as anti-apoptotic factor BCL-2 and so on (Sur et al., 2019).

## 4 MiRNAs Regulate DDR

MiRNAs loaded onto the RNA-induced silencing complex act on 3′-UTR region of mRNAs of DDR-related genes, so as to degenerate mRNA or produce translational silencing, thereby affecting the expression of genes and exert their biological function (Michlewski and Caceres, 2019; Majidinia et al., 2020). The role of miRNAs in DDR is elucidated from three aspects: miRNA′ regulation on cell cycle, DNA damage repair and apoptosis.

### 4.1 MiRNAs Regulate Cell Cycle

The cell cycle consists of G1 phase, S phase, G2 phase and M phase. In addition to these, cells in G0 phase are temporarily removed from the cell cycle and do not divide for the moment, but still have the potential to divide (Ferreira et al., 2020). Those cells will re-enter G1 phase and start the cell cycle when receiving a signal stimulus appropriately (Ferreira et al., 2020). When DDR occurs, cell cycle checkpoints are activated, which may result in cell cycle arrest and prevent damaged DNA from replicating. Cells in G1 to S and G2 to M phases are of particular importance, which undergo a complex series of molecular changes and are susceptible to environmental stimulation (Hossian et al., 2019). The miRNAs affect cell cycle mainly by regulating the progression of G0/G1, G1/S, and G2/M phase transitions.

#### 4.1.1 miRNAs Regulate G0/G1 Phase

Most miRNAs regulate cell cycle by inducing G0/G1 phase arrest. E2F transcription factors, cyclins and CDKs families play significant roles in cell cycle regulation (Huber et al., 2021). It can be seen from literatures that miRNAs induce G0/G1 phase arrest mainly by influence the expression of cell cycle regulators directly or indirectly. In cases of human glioma, overexpression of miR-320c dramatically down-regulated the ratio of S phase cells and blocked cell cycle at G1 phase by suppressing the expression of cyclin D1 and CDK6 (Lv et al., 2018). MiR-424-5p repressed cell proliferation and induced G0/G1 phase arrest by directly regulating its downstream gene E2F7 in hepatocellular carcinoma cells (Zhao et al., 2020). Moreover, Zhang *et al.*(Zhang et al., 2018) proved that miR-1258 was inhibited in colorectal cancer, and upregulation of miR-1258 blocked cell cycle at G1 phase *in vivo* and *in vitro*. Subsequently, miR-1258 was found to affect the expression of cyclin D1 and p21 through miR-1258s target E2F8 (Zhang et al., 2018). These results confirmed that miRNAs might influence multiple E2F transcription factor family members, thereby affecting the level of cyclins and CDK inhibitors and ultimately regulating G0/G1 phase. It is generally recognized that CDKs are cyclin-dependent kinases and affected by cyclins or CDK inhibitors such as p21. But surprisingly, the expression of CDK2 was not changed with cyclin D1 and p21 in colorectal cancer (Zhang et al., 2018). Therefore, more cell cycle-related CDKs expression should be detected to further determine that which CDK was indirectly affected by miR-1258 in colorectal cancer.

In addition to the typical cell cycle regulators mentioned above, some novel proteins have also been found to participate in the regulation of G0/G1 phase by miRNAs, enriching the regulatory network of miRNAs on cell cycle. For instance, triple negative breast cancer cells expressed high level of Neuropilin-1 (NRP-1) (Zhang et al., 2021). MiR-124-3p bound to 3′-UTR region of NRP-1 mRNA, blocked cell cycle at G0/G1 phase and inhibited cell proliferation (Zhang et al., 2021). After that, knockdown of NRP-1 increased the level of p27 and suppressed cyclin E and CDK2 (Zhang et al., 2021). It is not difficult to conjecture that the novel proteins may act as mediators between miRNAs and cell cycle, and affect the downstream cyclins, CDK inhibitors and CDKs family. Besides, kinesin family protein 22 (KIF22) is a kallikin-like DNA-binding protein that play a role in tumorigenesis (Wang et al., 2021). The KIF22 silencing resulted in an increase of G0/G1 phase esophageal squamous cells (Wang et al., 2021), leading to further study on the correlation between KIF22 and cell cycle. The binding of miR-122 to KIF22 was found and inhibition of miR-122 blocked the regulatory effect of si-KIF22 on p21, p27, cyclin D1 and CDK2 (Wang et al., 2021), suggested that these were all indirect downstream proteins of miR-122. Furthermore, chaperonin containing TCP1 subunit 3 (CCT3) is a component of chaperonin in eukaryotes, which has an impact on cell proliferation (Temiz et al., 2021). The mimic transfection of miR-24-3p, miR-128-3p, and miR-149-5p suppressed the level of CCT3 in ductal-breast adenocarcinoma and prostate carcinoma cells, which induced G0/G1 phase arrest and disturbed the mitochondrial membrane potential, eventually triggering apoptosis (Temiz et al., 2021). This result implied that three miRNAs above could not completely repair the DNA damages, hence inducing apoptosis. In summary, NRP-1 and KIF22 act as the novel downstream proteins of miRNA, and CCT3 is at least affected by miRNA, thus affecting the G0/G1 phase.

#### 4.1.2 miRNAs Induce G1 Phase Progression

Large numbers of miRNAs induce G1 phase arrest, whereas only a few miRNAs promote cell cycle by inducing G1 phase progression, which possibly because cells tend to suspend the cell cycle to prevent disordered cell replication when DDR occurs. For example, high concentration of mmu-miR-762 inhibitor arrested the cell cycle of mouse colon cancer cells at G0/G1 phase (Lai et al., 2021), suggesting that mmu-miR-762 might induce G1 phase progression. Zhao et al. (2019) found that under the infection of herpes simplex virus 2, miR-H4-5p bound to 3′-UTR of CDK inhibitor 2A and cyclin-dependent kinase-like 2 for negative regulation, to promote HeLa cells of S phase to the ratio of 41.8%, which was significantly higher than that in HeLa cells in control. In another study, transcriptomic sequencing of reticuloendotheliosis virus infected chicken embryo fibroblasts found that cyclin D1-CDK6 complex played a key role in the transition process from G1 phase to S phase (Zhai et al., 2018). KEGG enrichment analysis showed that target genes of a new miRNA, novel-72 was found to promote cell cycle and cell proliferation in this virus-infected cells (Zhai et al., 2018). From the reports so far, miRNAs might promote the transition from G1 phase to S phase in cells under the infection by virus, but more studies are needed to further elucidate that if the conjecture can be applied to more viruses.

#### 4.1.3 miRNAs Regulate G2/M Phase

A variety of miRNAs affect cell cycle by inducing G2 phase arrest, whereas whether miRNAs can promote G2 to M phase transition is rarely reported and not clear. MiR-191 has been reported increased in HeLa cells treated with cisplatin, an effective chemotherapeutic agent that induced DDR (Xu et al., 2020). Overexpression of miR-191 induced G2/M arrest via targeting chromosome condensation 2 regulator (RCC2). The inhibition of RCC2 mimicked the effects of miR-191 (Xu et al., 2020), which clarified the mechanism of miR-191 on cell cycle. Compared with that in healthy people, the level of miR-582-3p was decreased in blood of leukemia patients, and overexpression of miR-582-3p increased the ratio of G2/M phase cells in acute myeloid leukemia (Li et al., 2019). Cyclin B2 encoded by CCNB2 gene is a regulator of G2/M transition (Li et al., 2019). MiR-582-3p reduced the level of cyclin B2 via binding to CCNB2 mRNA, accompanied by its negative effects on CDK1 and cyclin B1 expression (Li et al., 2019). Similarly, miR-181c-5p blocked cell cycle at G2/M phase in injured osteoblasts via binding to 3′-UTR of cyclin B1 (Sun Z. et al., 2019). These studies confirmed that miRNAs regulated the proteins associated with G2 to M phase transition, including RCC2, cyclin B1 and cyclin B2. Moreover, a combinatorial treatment of miR-143 and miR-506 made cell cycle halt at G2/M phase transitions and suppressed the levels of CDK1, CDK4 and CDK6 in A549 non-small lung cancer cells (Hossian et al., 2019), suggested that miR-143 and miR-506 might induce G2 phase arrest via CDKs family, which revealed the potential of multiple miRNAs acting together on cell cycle.

### 4.2 MiRNAs Regulate DNA Damage Repair

DNA damages can be divided into SSBs and DSBs. SSBs are mainly repaired by BER and NER, while DSBs are processed through HR and NHEJ (Mota et al., 2019). BER recognize and repair the damage of a single base. When the DNA helix distortion is occurred, DNA SSBs are serious and be repaired by NER (Piotto et al., 2018). NHEJ mainly occurs in G0/G1 phase and directly connects the DNA end, so the risk of chromosomal translocations is increased (Pozzesi et al., 2014). In S and G2/M phases, HR is preferred and relies on homologous DNA chromosomes as templates for error-free repair of DNA (Piotto et al., 2018). Numbers of miRNAs regulate DDR by influencing different ways of DNA damage repair.

#### 4.2.1 Repair of DNA SSBs

Based on the fact that DNA double-strand damages cause the most serious consequences, most of the current studies on DNA damage repair focus on the repair of DSBs, and a few reports have demonstrated the regulatory role of miRNA in DNA SSBs. Poly (ADP-ribose) polymerase 1 (PARP-1) is a crucial DNA repair enzyme deeply involved in SSBs, and also related to DSBs (Yakovlev, 2015). The loss of PARP-1 activity leads to the SSBs accumulation, and SSBs may be converted to DSBs subsequently (Yakovlev, 2015). In human neuroblastoma cells, acute and chronic cocaine treatment decreased the level of miR-125b and increased the expression abundance of PARP-1, but did not change the level of cleaved-PARP-1 (Dash et al., 2017), implied that miR-125b was probably associated with DNA damage repair, and might have little effect on apoptosis marked by cleaved-PPAR-1. After that, the assays confirmed that miR-125b bound to 3′-UTR of PARP-1 mRNA (Dash et al., 2017). A few years later, another study used the luciferase reporter and pulldown assays found that miR-124 also directly bound to 3′-UTR of PARP-1 mRNA (Dash et al., 2020). Interestingly, the binding region of miR-124 and PARP-1 was partially same as the target sequence of miR-125b on PARP-1 (Dash et al., 2020), which was well demonstrated that a target gene can be regulated by several different miRNAs. Next, the titration experiment was carried out to assess the binding activity of two miRNAs to PARP-1, and showed that compared with miR-125b, miR-124 conferred a dominant post-transcriptional inhibition on PARP-1 (Dash et al., 2020). Therefore, in view of the same binding region, further investigation is needed to clarify that whether the sequence is essential for a miRNA binding with PARP-1 and whether other miRNAs containing this sequence have a negative effect on PARP-1. Subsequently, this sequence may serve as a novel and effective characteristic for screening for DNA SSBs related regulatory miRNAs. In addition to affecting PARP-1, miRNAs may also interfere with DNA SSBs in other way. Kang et al. (2019) found that miR-3912-5p might bind to 3′-UTR of BER protein NEIL2, thereby affecting DNA damage in lens cells. And miR-590 slowed the proliferation and promoted the repair of SSBs in embryonic stem cells (Liu et al., 2014), but the mechanism of which is still unclear.

#### 4.2.2 Repair of DNA DSBs

DSB is the most serious type of DNA damage, and its excessive accumulation leads to the genomic instability and may result in tumorigenesis. The formation of DSBs is often accompanied by phosphorylation of histone H2AX into γ-H2AX (Zeng et al., 2019). The way to repair DSBs depends on the cell cycle: NHEJ fixes DSBs mainly in G0/G1 phase, which is usually faster but prone to errors (Srinivasan et al., 2019). HR fixes DSBs in S and G2/M phases, which is slow and not prone to errors (Yakovlev, 2015; Piotto et al., 2018). In miR-34a-deficient hematopoietic stem cells, the content of γ-H2AX was increased, whereas the expression of DNA repair genes associated with HR and NHEJ was decreased (Zeng et al., 2019), determined that miR-34a had a positive effect on DDR. C-terminal binding protein interaction protein (CTIP) is an endonuclease, whose function is to recognizes damaged DNA specifically and participates in the HR process (Yang et al., 2019). The report showed that miR-130b facilitated the production of DSBs and subsequently promoted apoptosis by targeting CTIP in cervical cancer cells (Yang et al., 2019). It is commonly believed that BRCA1 and RAD17 proteins are associated with DDR, and the transcription inhibition of them leads to excessive build-up of DNA DSBs (Valenti et al., 2019). Experiments verified that the miR-205-5p/BRCA1/RAD17 axis increased the level of γ-H2AX and made DNA repair inefficient in head and neck squamous cell carcinomas *in vivo* and *in vitro* (Foray et al., 2003; Valenti et al., 2019), revealed that miR-205-5p/BRCA1/RAD17 axis had the potential to be a therapeutic target for cancer. Meanwhile, the value of miR-205-5p as a potential therapeutic agent needs to be further confirmed.

RAD51 is an extremely important HR regulator, which can be an indirect or direct downstream molecule of miRNAs to influence DNA DSBs repair. BRCA1 was found to directly bind to RAD51 (Zhao et al., 2017), which might be the upstream factor of RAD51 in DDR process. Meanwhile, RAD51 was negatively regulated by RING1 protein, which was a target of miR-3909 (Han et al., 2020). Moreover, all miR-34 family (miR-34a, miR-34b and miR-34c) could suppress the expression of RAD51 to inhibit HR process in human colon adenocarcinoma cells (Chen S. et al., 2019). This result provides solid evidence that the effects of miRNAs on DDR depend largely on the structure of miRNAs. Furthermore, Piotto et al. (2018) found that miR-96-5p targeted RAD51, whereas miR-874-3p targeted NHEJ related genes (PRKDC, LIG1) and affected the expression abundance of DNA-PKcs, leading to death of non-small lung cancer cells. The effects of miR-96-5p and miR-874-3p on DNA repair were similar to chemical inhibitors of HR and NHEJ process respectively (Piotto et al., 2018), which perfectly confirmed that miR-96-5p and miR-874-3p have their respective roles in the repair of DNA DSBs. In addition, γ-ray irradiation increased the level of miR-1246 in exosomes and treating unirradiated cells with this exosome reduced the activity of NHEJ via miR-1246′ binding with 3′-UTR of LIG4 (Mo et al., 2018). The result confirmed that exogenous stimulation could alter the content of a miRNA in exosomes, and raised the possibility that the delivery of miRNAs by exosomes might be a potential therapeutic approach for diseases by influencing the DDR.

### 4.3 MiRNAs Regulate Apoptosis

Apoptosis is an orderly cell death mode controlled by genes in order to maintain the homeostasis of cell internal environment (Chen Z. et al., 2019). It mainly includes mitochondria-mediated intrinsic apoptosis pathway and death receptor-mediated extrinsic apoptosis pathway (Chen Z. et al., 2019; Jiang et al., 2020).

#### 4.3.1 miRNAs Promote Apoptosis

In previous studies, the positive effects of miRNAs on apoptosis have been reported, and the downstream genes of miRNAs include BCL-2 family (pro-apoptotic gene Bax, Bim and anti-apoptotic gene Bcl-2, etc.), Fas-associated phosphatase 1 (Fap-1), Caspase family and new molecules associated with apoptosis. In prostate cancer cells, overexpression of miR-29b upregulated the expression of Bim, induced the pro-apoptotic factor cytochrome C and cleaved-PARP-1 release, leading to apoptosis in a time-dependent manner (Agarwal et al., 2009; Sur et al., 2019). Death receptor-mediated apoptosis pathway is activated by Fas interacting with Fas-associated protein with death domain (FADD). After that, the Caspases family was activated (Pozzesi et al., 2014). MiR-200c-3p was believed to link with endothelial function via its target Fap-1, and Fap-1 is an inhibitor of Fas (Jiang et al., 2020). As a result of miR-200c-3p overexpression, Fap-1 was inhibited and Fas was activated, leading to increased FADD, cleaved-caspase-8 and cleaved-caspase-3. These effects were abrogated by miR-200c-3p inhibitor alone or co-use of miR-200c-3p inhibitor and siRNA of Fap-1 (Jiang et al., 2020), which is well confirmed that miR-200c-3p significantly promoted the death receptor-mediated apoptosis pathway.

A recent study showed that the expression of miR-181c-5p was up-regulated in the early stage of myocardial infarction, whose target gene is protein tyrosine phosphatase nonreceptor type 4 (PTPN4) (Ge et al., 2019). As a result, PTPN4 silencing elevated the Bax/Bcl-2 ratio, the abundance of cleaved-caspases 3 and produced more TUNEL positive cells in cardiomyocytes, which exacerbated mitochondria-mediated apoptosis and mimicking the pro-apoptotic effect of miR-181c-5p overexpression (Ge et al., 2019), confirming that PTPN4 is a new molecule involved in apoptosis. As mentioned above, CCT3 is a target of miR-24-3p and miR-128-3p. In ductal-breast adenocarcinoma cells, the expression of BAX was increased via suppressing CCT3 by miR-24-3p or miR-128-3p (Temiz et al., 2021). Besides, miR-24-3p transfection induced the release of cytochrome C and then enhanced the activity of initiator and effector caspase (caspase 9 and 3). And miR-128-3p did not affect cytochrome C release, but increased the proportion of late apoptotic cells and also enhanced the caspase activity (caspase 8,9 and 3) (Temiz et al., 2021). Therefore, miR-128-3p may have stronger binding activity to CCT3 gene than miR-24-3p, thus leading to more characteristics of late apoptosis. In this study, miR-128-3p influenced BAX and BCL-2 located in mitochondria, as well as caspase 8 in the exogenous apoptosis pathway, demonstrating that miR-128-3p simultaneously regulate both mitochondria-dependent and mitochondria-independent apoptosis. To sum up, several miRNAs affect apoptotic participants and promote apoptosis by targeting Fap-1, PTPN4 and CCT3. But most of the published reports only found the causal relationship between miRNAs and apoptosis, and the specific targets of miRNAs involved in apoptosis need to be further investigated.

#### 4.3.2 miRNA Inhibits Apoptosis

The miRNAs not only take part in the promotion of apoptosis, but also have a link with the inhibition of apoptosis. PDCD4 is a tumor suppressor, and its knockdown activated the transcription factor AP-1 (Song et al., 2019). MiR-21 directly inhibited apoptosis by the miR-21/PDCD4/AP-1 axis in infarcted cardiomyocytes (Song et al., 2019). Besides, the application of exogenous miR-21 significantly reduced apoptosis and improved cardiac function in preclinical myocardial infarction mice (Song et al., 2019), suggested that miR-21 might be a potential agent to treat myocardial infarction. Similarly, the expression of miR-21 was decreased in myocardial injured rats. Overexpression of miR-21 reduced the Bax/Bcl-2 ratio and the level of caspase-3 by inhibiting TLR4/nuclear factor-κB (NF-κB) pathway (Pan et al., 2018), leading to the reduced level of apoptosis in rat. Moreover, miR-26a-5p activated the PI3K/protein kinase B (AKT) pathway via targeting phosphate and tensin homology deleted on chromosome 10, which inhibited apoptosis of endothelial cells in coronary artery mice (Jing et al., 2019). Furthermore, the expression of miR-27a was suppressed in vascular endothelium. Inhibition of miR-27a promoted the expression of FADD (Sun Y. et al., 2019), confirming that miR-27a inhibited apoptosis mediated by death receptor pathway. Subsequently, luciferase reporter assay verified that miR-27a inhibited apoptosis via binding to 3′-UTR of FADD (Sun Y. et al., 2019). In summary, multiple signaling pathways are associated with the inhibition of apoptosis by miRNAs, such as TLR4/NF-κB and PI3K/AKT pathway, but the targets of miRNAs among the process have not been elucidated yet.

#### 4.3.3 The Dual Roles of miRNAs in Apoptosis

Some miRNAs play dual roles in the process of apoptosis, and the effect of a miRNA on apoptosis may be cell-type specific or disease-specific. MiR-7 exerts dual effects on apoptosis in different tissues under stress conditions. There is a report showed that miR-7 inhibited mitogen-activated protein kinase kinase (MAPKK)/extracellular regulated protein kinase (ERK) and NF-κB pathways by directly targeting MAPKK kinase 9, leading to the upregulation of apoptosis in pancreatic cancer cells (Xia et al., 2018). Another report of benign prostatic hyperplasia epithelial cells and human prostate epithelial cells also suggested that miR-7 had a promoting effect on apoptosis which was related to PI3K/AKT and Ras-associated factor-1/ERK 1/2 pathways (Han et al., 2019). However, miR-7 inhibitor increased apoptosis in colorectal cancer cells (Mo et al., 2019), suggested that miR-7 might have a negative effect on apoptosis. As mentioned above, miR-181c-5p elevated the level of Bax/Bcl-2 ratio and cleaved-caspases 3, showed a pro-apoptotic effect in cardiomyocytes (Ge et al., 2019). Nevertheless, miR-181c-5p acted on 3′-UTR region of HMGB1 mRNA and inhibited apoptosis in microglia (Li et al., 2021). Compared with miR-181c-5p treatment, the overexpression of HMGB1 showed the adverse effect and enhanced apoptosis (Li et al., 2021), confirming the anti-apoptotic role of miR-181c-5p. From the above, MAPKK/ERK, NF-κB, PI3K/AKT and Ras-associated factor-1/ERK 1/2 signaling pathways are all linked with the regulation of miR-7 on apoptosis. These opposite effects of miR-7 and miR-181c-5p may be caused by differences in cell type and organ function.

## 5 Conclusion and Perspectives

**FIGURE 1:**
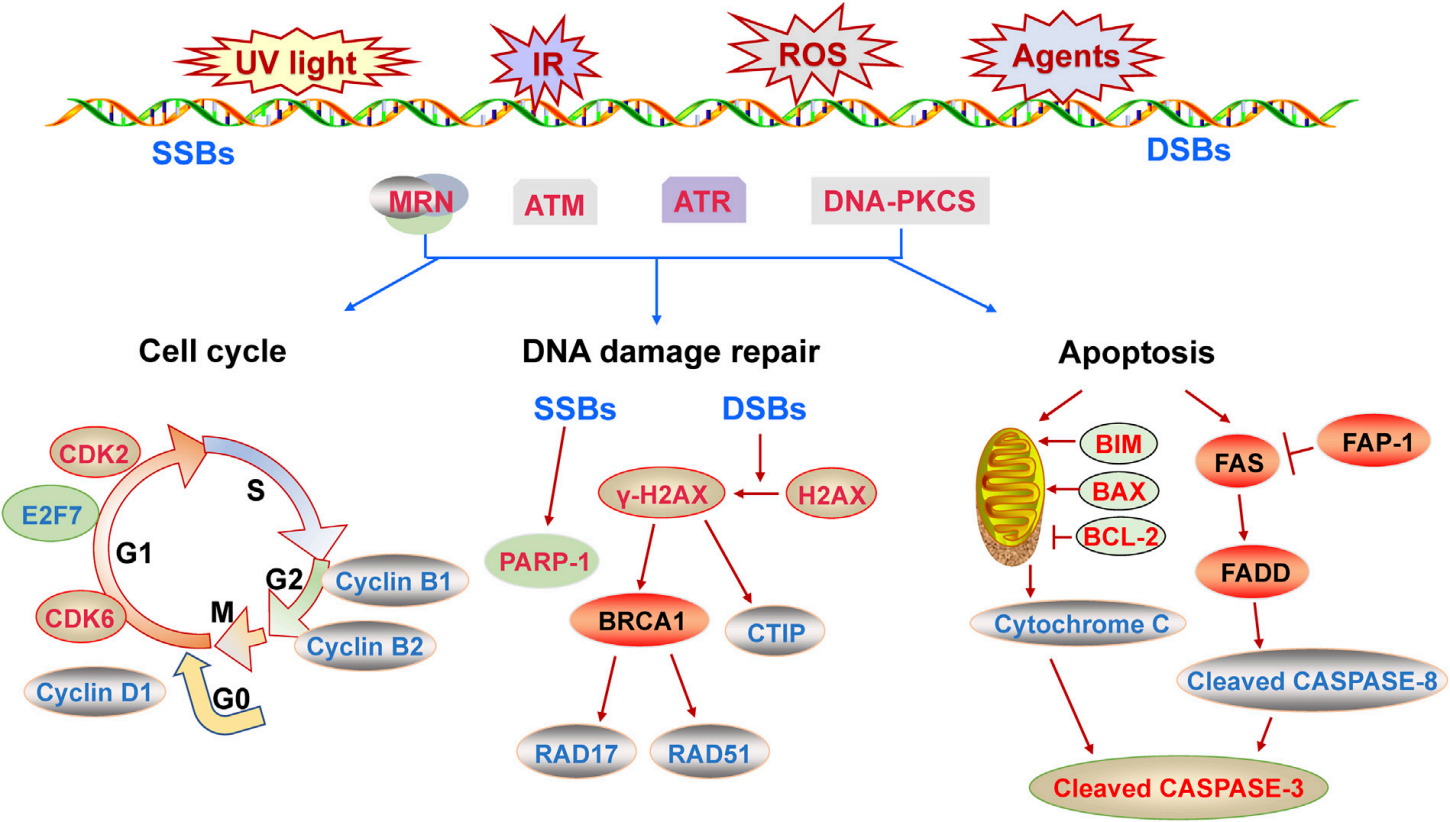
The molecular mechanism of DNA damage response described from three aspects of cell cycle, DNA repair and apoptosis.

**FIGURE 2:**
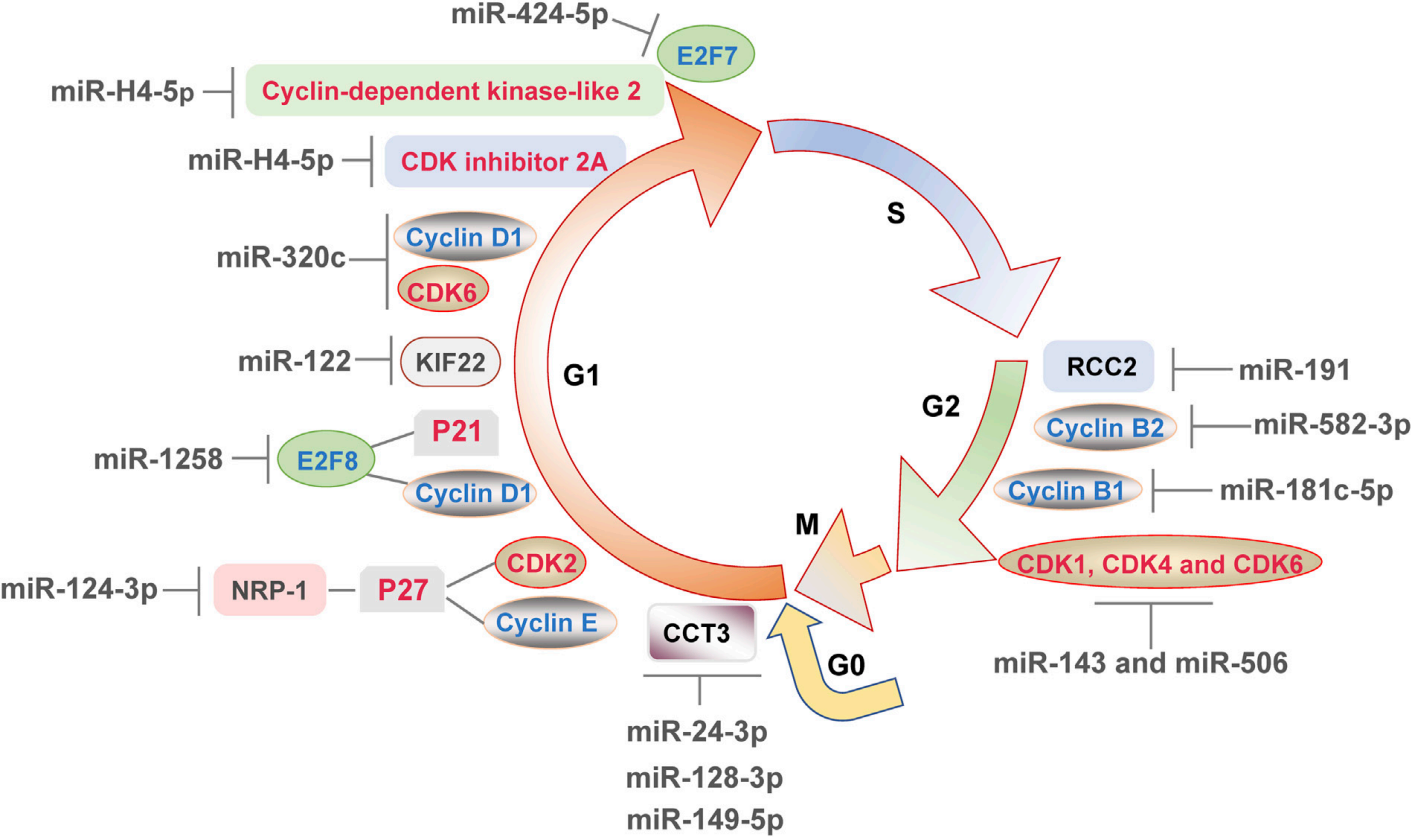
MicroRNAs directly or indirectly affect the expression of various components involved in the cell cycle, leading to cell cycle progression or arrest and finally regulating the DNA damage response.

**FIGURE 3:**
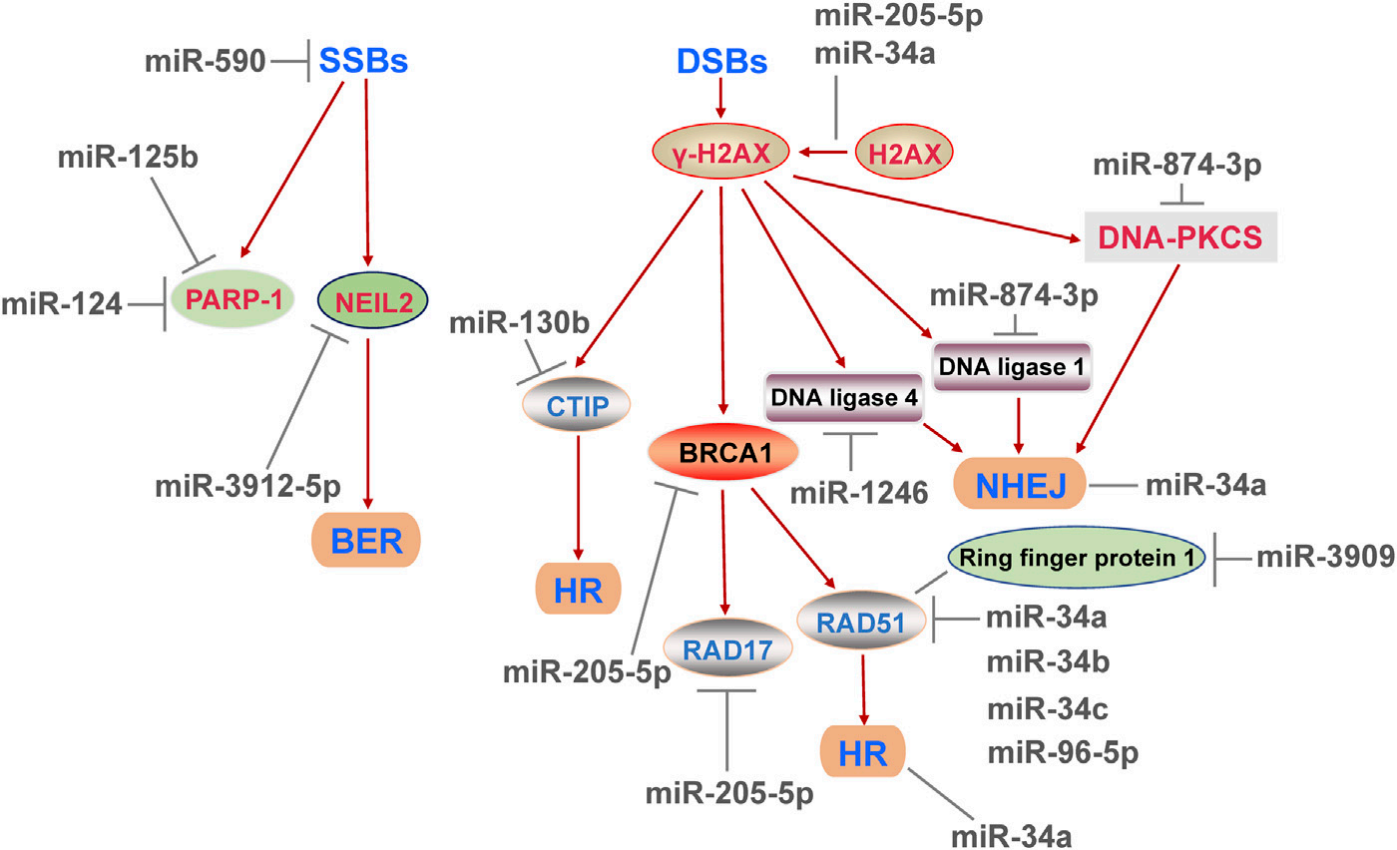
MicroRNAs affect various DNA repair pathways at least includes base excision repair, homologous recombination repair and non-homologous end joining repair, and regulate the repair efficiency of DNA single-strand and double-strand breaks, and ultimately have an impact on DNA damage response.

**FIGURE 4:**
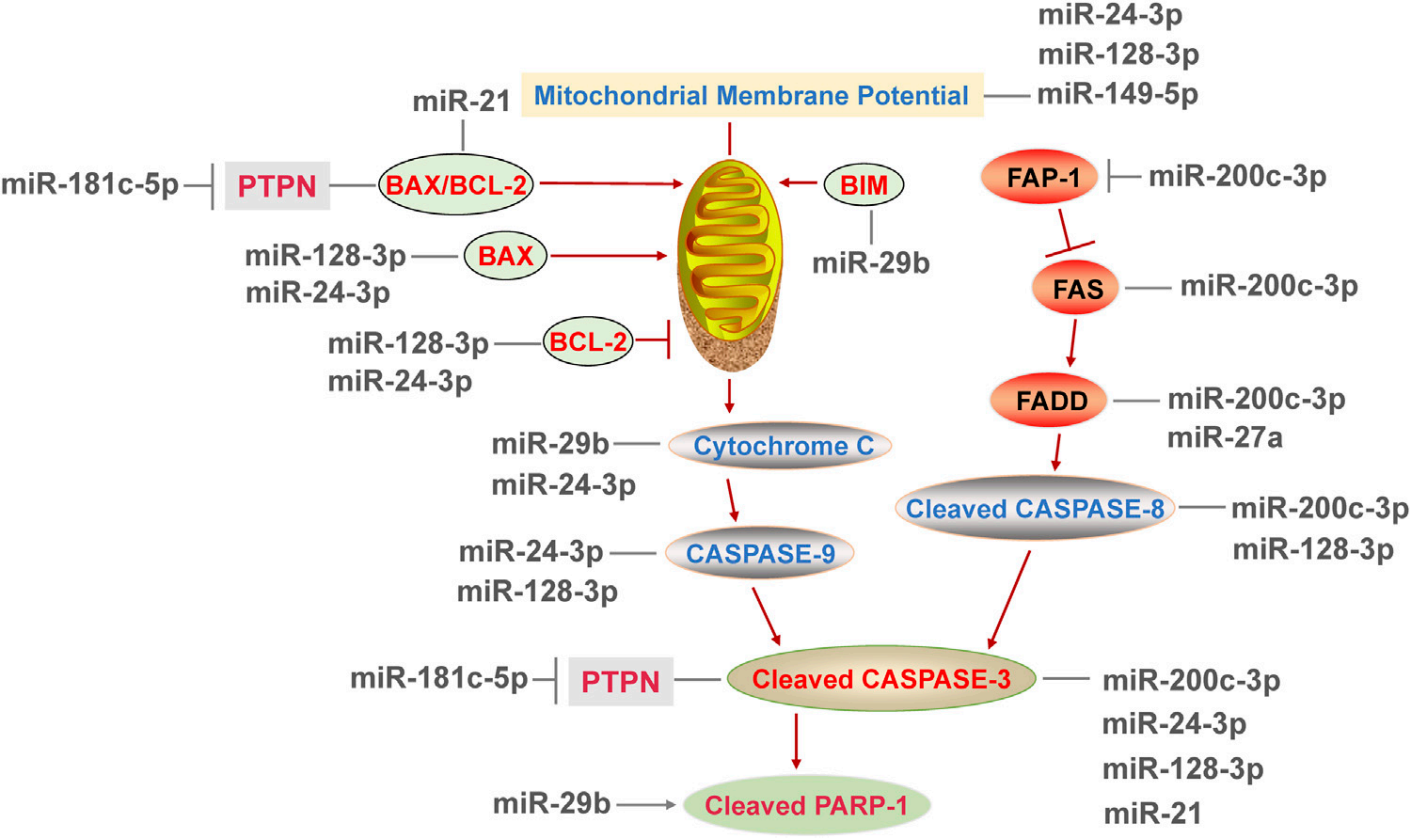
MicroRNAs regulate the DNA damage response by affecting the participators of both mitochondria-mediated intrinsic apoptosis pathway and death receptor-mediated extrinsic apoptosis pathway.

Overall, functional single-stranded miRNAs are formed by transcription, processing and maturation processes. Mature miRNAs induce the degradation of mRNA of target genes or inhibit their translation, regulating a variety of biological processes. The DDR regulates cell cycle, induces DNA damage repair and even causes apoptosis, and is closely related to cancers and other diseases. The mechanism of DDR is shown in Figure 1. MiRNAs can positively or negatively regulate DDR by inhibiting the expression of pivotal genes in the process of DDR. The role of miRNAs in DDR is elucidated from three aspects including cell cycle, DNA damage repair and apoptosis (Figures 2–4), and the target genes of miRNAs in DDR is shown in Table 1.

**TABLE 1:** The roles of microRNAs and their targets in DNA damage response.

MicroRNAs	Targets	Functions
miR-320c	Cyclin D1	G1 phase arrest
miR-320c	CDK6	G1 phase arrest
miR-424-5p	E2F7	G0/G1 phase arrest
miR-1258	E2F8	G1 phase arrest
MiR-124-3p	NRP-1	G0/G1 phase arrest
miR-122	KIF22	G0/G1 phase arrest
miR-H4-5p	CDK inhibitor 2A	G1 phase progression
miR-H4-5p	Cyclin-dependent kinase-like 2	G1 phase progression
miR-191	RCC2	G2/M phase arrest
miR-582-3p	Cyclin B2	G2/M phase arrest
miR-181-5p	Cyclin B1	G2/M phase arrest
miR-125b	PARP-1	SSBs accumulation
miR-124	PARP-1	SSBs accumulation
miR-3912-5p	NEIL2	SSBs accumulation
miR-130b	CTIP	DSBs accumulation
miR-205-5p	BRCA1	DSBs accumulation
miR-3909	RING1	DSBs accumulation
miR-34a	RAD51	DSBs accumulation
miR-34b	RAD51	DSBs accumulation
miR-34c	RAD51	DSBs accumulation
miR-96-5p	RAD51	DSBs accumulation
miR-874-3p	PRKDC	DSBs accumulation
miR-874-3p	LIG1	DSBs accumulation
miR-1246	LIG4	DSBs accumulation
miR-181-5p	PTPN4	Promote apoptosis
miR-200c-3p	Fap-1	Promote apoptosis
miR-21	PDCD4	Inhibit apoptosis
miR-26a-5p	Phosphate and tensin homology deleted on chromosome 10	Inhibit apoptosis
miR-27a	FADD	Inhibit apoptosis
miR-7	MAPKK kinase 9	Promote apoptosis
miR-181-5p	HMGB1	Inhibit apoptosis

On this basis, the following research directions can be considered in the future. 1) Numbers of miRNAs targeted cell cycle and DNA damage repair related genes. And there were many reports showed that the effects of miRNAs on apoptosis were dependent on the signaling pathways, but only a few targets of miRNAs involved in apoptosis were found. Therefore, more exploration in-depth is needed to confirm that whether this pattern is common and to determine its specific reasons. 2) The levels of miRNAs were decreased in multiple tumor cases, and exogenous stimulation increased the level of miRNAs in exosomes (Mo et al., 2018). Therefore, the relationships between miRNA levels of exosomes and tumorigenesis, progression or prognosis should be further studied, to determine the potential application value of miRNAs as biomarkers for early diagnosis of tumors. 3) The miRNAs significantly affect DDR and are closely related to tumorigenesis. However, there are still limitations in the way of exogenous miRNA delivering, which hinder the application of miRNAs. Extracellular vesicles (micro-vesicles and exosomes) are secreted vesicles with membrane structures, which protect the contents from degradation and enter the recipient cell. Extracellular vesicles have been reported as a novel strategy for the exogenous addition of miRNAs (Song et al., 2019; Chaparro et al., 2021). It is necessary to explore the effects of miRNAs delivered by extracellular vesicle on diseases and promote the clinical application of miRNAs on tumors and other diseases.

In summary, this review elucidates the regulatory mechanism of miRNAs on DDR, which is conducive to providing potential biomarkers for DDR-related diseases and providing a new direction for the prevention, early screening and treatment of cancers.

## Abbreviations

AKT, protein kinase B; ATM, ataxia telangiectasia mutated; ATR, ATM- and Rad-3-related protein; BAX, BCL-2-Associated X; BCL-2, B cell lymphoma 2; BER, base excision repair; BIM, BCL-2-interacting mediator of cell death; CCT3, chaperonin containing TCP1 subunit 3; CDK, cyclin-dependent kinase; DSBs, double-strand breaks; CTIP, C-terminal binding protein interacting protein; FADD, Fas-associated protein with death domain; Fap-1, Fas-associated phosphatase 1; Fas, TNF receptor superfamily member 6; HR, homologous recombination repair; KIF22, kinesin family protein 22; MAPKK, mitogen-activated protein kinase kinase; NER, nucleotide excision repair; NHEJ, non-homologous end joining repair; NRP-1, neuropilin-1; NF-κB, nuclear factor-κB; p21, cyclin-dependent kinase inhibitor 1A; p27, CDK inhibitor 1B; PARP1, poly (ADP-ribose) polymerase 1; PI3K, phosphatidylinositol-3-kinase; PTPN4, protein tyrosine phosphatase nonreceptor type 4; SSBs, single-strand breaks; UTR, untranslated region.

## References

[B1] Agarwal A. Mahfouz R. Z. Sharma R. K. Sarkar O. Mangrola D. Mathur P. P. (2009). Potential Biological Role of Poly (ADP-Ribose) Polymerase (PARP) in Male Gametes. Reprod. Biol. Endocrinol. 7, 143. 10.1186/1477-7827-7-143 19961617 PMC2800114

[B2] Chaparro A. Atria P. Realini O. Monteiro L. J. Betancur D. Acuña‐Gallardo S. (2021). Diagnostic Potential of Peri‐Implant Crevicular Fluid microRNA‐21‐3p and microRNA‐150‐5p and Extracellular Vesicles in Peri‐Implant Diseases. J. Periodontol. 92 (6), 11–21. 10.1002/JPER.20-0372 33185898

[B3] Chen S. Liu R. Wang Q. Qi Z. Hu Y. Zhou P. (2019a). MiR-34s Negatively Regulate Homologous Recombination through Targeting RAD51. Arch. Biochem. Biophys. 666, 73–82. 10.1016/j.abb.2019.03.017 30951682

[B4] Chen Z. Lai H. Hou L. Chen T. (2019b). Rational Design and Action Mechanisms of Chemically Innovative Organoselenium in Cancer Therapy. Chem. Commun. 56 (2), 179–196. 10.1039/c9cc07683b 31782422

[B5] Chou J. Quigley D. A. Robinson T. M. Feng F. Y. Ashworth A. (2020). Transcription-Associated Cyclin-dependent Kinases as Targets and Biomarkers for Cancer Therapy. Cancer Discov. 10 (3), 351–370. 10.1158/2159-8290.CD-19-0528 32071145

[B6] Dash S. Balasubramaniam M. Martínez-Rivera F. J. Godino A. Peck E. G. Patnaik S. (2020). Cocaine-regulated microRNA miR-124 Controls Poly (ADP-Ribose) Polymerase-1 Expression in Neuronal Cells. Sci. Rep. 10 (1), 11197. 10.1038/s41598-020-68144-6 32641757 PMC7343862

[B7] Dash S. Balasubramaniam M. Rana T. Godino A. Peck E. G. Goodwin J. S. (2017). Poly (ADP-Ribose) Polymerase-1 (PARP-1) Induction by Cocaine Is post-transcriptionally Regulated by miR-125b. eNeuro 4 (4), 17. 10.1523/ENEURO.0089-17.2017 PMC5562297 28828398

[B8] Fang Z. Pan Z. (2019). Essential Role of Ubiquitin-fold Modifier 1 Conjugation in DNA Damage Response. DNA Cel. Biol. 38 (10), 1030–1039. 10.1089/dna.2019.4861 31368785

[B9] Ferreira I. A. T. M. Porterfield J. Z. Gupta R. K. Mlcochova P. (2020). Cell Cycle Regulation in Macrophages and Susceptibility to HIV-1. Viruses 12 (8), 839. 10.3390/v12080839 PMC7472357 32751972

[B10] Foray N. Marot D. Gabriel A. Randrianarison V. Carr A. M. Perricaudet M. (2003). A Subset of ATM- and ATR-Dependent Phosphorylation Events Requires the BRCA1 Protein. EMBO J. 22 (11), 2860–2871. 10.1093/emboj/cdg274 12773400 PMC156770

[B11] Ge L. Cai Y. Ying F. Liu H. Zhang D. He Y. (20192019). miR-181c-5p Exacerbates Hypoxia/Reoxygenation-Induced Cardiomyocyte Apoptosis via Targeting PTPN4. Oxidative Med. Cell Longevity 2019, 1–15. 10.1155/2019/1957920 PMC6501226 31178952

[B12] Haferlach C. Bacher U. Kohlmann A. Schindela S. Alpermann T. Kern W. (2011). CDKN1B, Encoding the Cyclin-Dependent Kinase Inhibitor 1B (P27), Is Located in the Minimally Deleted Region of 12p Abnormalities in Myeloid Malignancies and its Low Expression Is a Favorable Prognostic Marker in Acute Myeloid Leukemia. Haematologica 96 (6), 829–836. 10.3324/haematol.2010.035584 21422114 PMC3105644

[B13] Han N. Zhang B. Wei X. Yu L. (2019). The Inhibitory Function of Icariin in Cell Model of Benign Prostatic Hyperplasia by Upregulation of miR‐7. Biofactors. 10.1002/biof.1591

[B14] Han T. Jing X. Bao J. Zhao L. Zhang A. Miao R. (2020). *H. pylori* Infection Alters Repair of DNA Double-Strand Breaks via SNHG17. J. Clin. Invest. 130 (7), 3901–3918. 10.1172/JCI125581 32538894 PMC7324211

[B15] Hossian A. K. M. N. Muthumula C. M. R. Sajib M. S. Tullar P. E. Stelly A. M. Briski K. P. (2019). Analysis of Combinatorial miRNA Treatments to Regulate Cell Cycle and Angiogenesis. JoVE 30 (145). 10.3791/59460 30985742

[B16] Huber K. Mestres‐Arenas A. Fajas L. Leal‐Esteban L. C. (2021). The Multifaceted Role of Cell Cycle Regulators in the Coordination of Growth and Metabolism. FEBS J. 288 (12), 3813–3833. 10.1111/febs.15586 33030287 PMC8359344

[B17] Jackson S. P. Bartek J. (2009). The DNA-Damage Response in Human Biology and Disease. Nature 461 (7267), 1071–1078. 10.1038/nature08467 19847258 PMC2906700

[B18] Jiang Y. Yang Y. Zhang C. Huang W. Wu L. Wang J. (2020). Upregulation of miR-200c-3p Induced by NaF Promotes Endothelial Apoptosis by Activating Fas Pathway. Environ. Pollut. 266 (1), 115089. 10.1016/j.envpol.2020.115089 32629210

[B19] Jing R. Zhong Q. Q. Long T. Y. Pan W. Qian Z. X. (2019). Downregulated miRNA-26a-5p Induces the Apoptosis of Endothelial Cells in Coronary Heart Disease by Inhibiting PI3K/AKT Pathway. Eur. Rev. Med. Pharmacol. Sci. 23 (11), 4940–4947. 10.26355/eurrev_201906_18084 31210329

[B20] Kang L. Zou X. Zhang G. Xiang J. Wang Y. Yang M. (2019). A Variant in a microRNA Binding Site in NEIL2 3′UTR Confers Susceptibility to Age‐Related Cataracts. FASEB J. 33 (9), 10469–10476. 10.1096/fj.201802291R 31253066

[B21] Kopp B. Khoury L. Audebert M. (2019). Validation of the γH2AX Biomarker for Genotoxicity Assessment: a Review. Arch. Toxicol. 93 (8), 2103–2114. 10.1007/s00204-019-02511-9 31289893

[B22] Lai P.-S. Chang W.-M. Chen Y.-Y. Lin Y.-F. Liao H.-F. Chen C.-Y. (2021). Circulating microRNA-762 Upregulation in Colorectal Cancer May Be Accompanied by Wnt-1/β-Catenin Signaling. Cbm 32 (2), 111–122. 10.3233/CBM-203002 PMC12500045 34092606

[B23] Lee R. C. Feinbaum R. L. Ambros V. (1993). The *C. elegans* Heterochronic Gene Lin-4 Encodes Small RNAs with Antisense Complementarity to Lin-14. Cell 75 (5), 843–854. 10.1016/0092-8674(93)90529-y 8252621

[B24] Li H. Tian X. Wang P. Huang M. Xu R. Nie T. (2019). MicroRNA-582-3p Negatively Regulates Cell Proliferation and Cell Cycle Progression in Acute Myeloid Leukemia by Targeting Cyclin B2. Cell Mol. Biol. Lett. 24, 66. 10.1186/s11658-019-0184-7 31844417 PMC6894134

[B25] Li M. Hu J. Peng Y. Li J. Ren R. (2021). CircPTK2-miR-181c-5p-HMGB1: a New Regulatory Pathway for Microglia Activation and Hippocampal Neuronal Apoptosis Induced by Sepsis. Mol. Med. 27 (1), 45. 10.1186/s10020-021-00305-3 33952191 PMC8101146

[B26] Liu Q. Wang G. Chen Y. Li G. Yang D. Kang J. (2014). A miR-590/Acvr2a/Rad51b Axis Regulates DNA Damage Repair During mESC Proliferation. Stem Cel. Rep. 3 (6), 1103–1117. 10.1016/j.stemcr.2014.10.006 PMC4264031 25458897

[B27] Lv Q.-L. Zhu H.-T. Li H.-M. Cheng X.-H. Zhou H.-H. Chen S.-H. (2018). Down-Regulation of miRNA-320c Promotes Tumor Growth and Metastasis and Predicts Poor Prognosis in Human Glioma. Brain Res. Bull. 139, 125–132. 10.1016/j.brainresbull.2018.02.009 29438779

[B28] Majidinia M. Mir S. M. Mirza-Aghazadeh-Attari M. Asghari R. Kafil H. S. Safa A. (2020). MicroRNAs, DNA Damage Response and Ageing. Biogerontology 21 (3), 275–291. 10.1007/s10522-020-09862-2 32067137

[B29] Majidinia M. Yousefi B. (2016). DNA Damage Response Regulation by microRNAs as a Therapeutic Target in Cancer. DNA Repair 47, 1–11. 10.1016/j.dnarep.2016.09.003 27697364

[B30] McManus M. T. (2003). MicroRNAs and Cancer. Semin. Cancer Biol. 13 (4), 253–258. 10.1016/s1044-579x(03)00038-5 14563119

[B31] Michlewski G. Cáceres J. F. (2019). Post-Transcriptional Control of miRNA Biogenesis. RNA 25 (1), 1–16. 10.1261/rna.068692.118 30333195 PMC6298569

[B32] Mo D. Liu W. Li Y. Cui W. (2019). Long Non-Coding RNA Zinc Finger Antisense 1 (ZFAS1) Regulates Proliferation, Migration, Invasion, and Apoptosis by Targeting miR-7-5p in Colorectal Cancer. Med. Sci. Monit. 25, 5150–5158. 10.12659/MSM.916619 31295229 PMC6640168

[B33] Mo L.-J. Song M. Huang Q.-H. Guan H. Liu X.-D. Xie D.-F. (2018). Exosome-Packaged miR-1246 Contributes to Bystander DNA Damage by Targeting LIG4. Br. J. Cancer 119 (4), 492–502. 10.1038/s41416-018-0192-9 30038324 PMC6134031

[B34] Mota M. B. S. Carvalho M. A. Monteiro A. N. A. Mesquita R. D. (2019). DNA Damage Response and Repair in Perspective: *Aedes aegypti*, *Drosophila melanogaster* and *Homo sapiens* . Parasites Vectors 12 (1), 533. 10.1186/s13071-019-3792-1 31711518 PMC6849265

[B35] Ou H.-L. Schumacher B. (2018). DNA Damage Responses and P53 in the Aging Process. Blood 131 (5), 488–495. 10.1182/blood-2017-07-746396 29141944 PMC6839964

[B36] Pan Y. Q. Li J. Li X. W. Li Y. C. Li J. Lin J. F. (2018). Effect of miR-21/tlr4/nf-Κb Pathway on Myocardial Apoptosis in Rats with Myocardial Ischemia-Reperfusion. Eur. Rev. Med. Pharmacol. Sci. 22 (22), 7928–7937. 10.26355/eurrev_201811_16420 30536340

[B37] Piotto C. Biscontin A. Millino C. Mognato M. (2018). Functional Validation of miRNAs Targeting Genes of DNA Double-Strand Break Repair to Radiosensitize Non-Small Lung Cancer Cells. Biochim. Biophys. Acta (Bba) - Gene Regul. Mech. 1861 (12), 1102–1118. 10.1016/j.bbagrm.2018.10.010 30389599

[B38] Pozzesi N. Fierabracci A. Liberati A. M. Martelli M. P. Ayroldi E. Riccardi C. (2014). Role of Caspase-8 in Thymus Function. Cell Death Differ. 21 (2), 226–233. 10.1038/cdd.2013.166 24270406 PMC3890959

[B39] Singh R. Letai A. Sarosiek K. (2019). Regulation of Apoptosis in Health and Disease: the Balancing Act of BCL-2 Family Proteins. Nat. Rev. Mol. Cel. Biol. 20 (3), 175–193. 10.1038/s41580-018-0089-8 PMC7325303 30655609

[B40] Song Y. Zhang C. Zhang J. Jiao Z. Dong N. Wang G. (2019). Localized Injection of miRNA-21-Enriched Extracellular Vesicles Effectively Restores Cardiac Function after Myocardial Infarction. Theranostics 9 (8), 2346–2360. 10.7150/thno.29945 31149048 PMC6531307

[B41] Srinivasan G. Williamson E. A. Kong K. Jaiswal A. S. Huang G. Kim H.-S. (2019). MiR223-3p Promotes Synthetic Lethality in BRCA1-Deficient Cancers. Proc. Natl. Acad. Sci. U.S.A. 116 (35), 17438–17443. 10.1073/pnas.1903150116 31395736 PMC6717293

[B42] Sun Y. Xiao Y. Sun H. Zhao Z. Zhu J. Zhang L. (2019a). miR-27a Regulates Vascular Remodeling by Targeting Endothelial Cells' Apoptosis and Interaction with Vascular Smooth Muscle Cells in Aortic Dissection. Theranostics 9 (25), 7961–7975. 10.7150/thno.35737 31695809 PMC6831472

[B43] Sun Z. Li Y. Wang H. Cai M. Gao S. Liu J. (2019b). miR‐181c‐5p Mediates Simulated Microgravity‐induced Impaired Osteoblast Proliferation by Promoting Cell Cycle Arrested in the G 2 Phase. J. Cel Mol Med 23 (5), 3302–3316. 10.1111/jcmm.14220 PMC6484313 30761733

[B44] Sur S. Steele R. Shi X. Ray R. B. (2019). miRNA-29b Inhibits Prostate Tumor Growth and Induces Apoptosis by Increasing Bim Expression. Cells 8 (11), 1455. 10.3390/cells8111455 PMC6912792 31752117

[B45] Temiz E. Koyuncu İ. Sahin E. (2021). CCT3 Suppression Prompts Apoptotic Machinery through Oxidative Stress and Energy Deprivation in Breast and Prostate Cancers. Free Radic. Biol. Med. 165, 88–99. 10.1016/j.freeradbiomed.2021.01.016 33508424

[B46] Valenti F. Sacconi A. Ganci F. Grasso G. Strano S. Blandino G. (2019). The miR-205-5p/BRCA1/RAD17 Axis Promotes Genomic Instability in Head and Neck Squamous Cell Carcinomas. Cancers 11 (9), 1347. 10.3390/cancers11091347 PMC6771082 31514456

[B47] Van Opdenbosch N. Lamkanfi M. (2019). Caspases in Cell Death, Inflammation, and Disease. Immunity 50 (6), 1352–1364. 10.1016/j.immuni.2019.05.020 31216460 PMC6611727

[B48] Wang J. Yu P. Y. Yu J. P. Luo J. D. Sun Z. Q. Sun F. (2021). KIF22 Promotes Progress of Esophageal Squamous Cell Carcinoma Cells and Is Negatively Regulated by miR-122. Am. J. Transl. Res. 13 (5), 4152–4166. 34150005 PMC8205736

[B49] Wijnhoven B. P. L. Michael M. Z. Watson D. I. (2007). MicroRNAs and Cancer. Br. J. Surg. 94 (1), 23–30. 10.1002/bjs.5673 17205498

[B50] Xia J. Cao T. Ma C. Shi Y. Sun Y. Wang Z. P. (2018). miR-7 Suppresses Tumor Progression by Directly Targeting MAP3K9 in Pancreatic Cancer. Mol. Ther. - Nucleic Acids 13, 121–132. 10.1016/j.omtn.2018.08.012 30290304 PMC6171162

[B51] Xu X. Zhou X. Zhang J. Li H. Cao Y. Tan X. (2020). MicroRNA‐191 Modulates Cisplatin‐Induced DNA Damage Response by Targeting RCC2. FASEB J. 34 (10), 13573–13585. 10.1096/fj.202000945R 32803782

[B52] Yakovlev V. A. (2015). Nitric Oxide: Genomic Instability and Synthetic Lethality. Redox Biol. 5, 414. 10.1016/j.redox.2015.09.013 28162271

[B53] Yang L. Yang B. Wang Y. Liu T. He Z. Zhao H. (2019). The CTIP‐Mediated Repair of TNF‐α‐induced DNA Double‐Strand Break Was Impaired by miR‐130b in Cervical Cancer Cell. Cell Biochem. Funct. 37 (7), 534–544. 10.1002/cbf.3430 31418900 PMC6852181

[B54] Yue X. Bai C. Xie D. Ma T. Zhou P.-K. (2020). DNA-PKcs: A Multi-Faceted Player in DNA Damage Response. Front. Genet. 11, 607428. 10.3389/fgene.2020.607428 33424929 PMC7786053

[B55] Zeng H. Hu M. Lu Y. Zhang Z. Xu Y. Wang S. (2019). MicroRNA 34a Promotes Ionizing Radiation-Induced DNA Damage Repair in Murine Hematopoietic Stem Cells. FASEB j. 33 (7), 8138–8147. 10.1096/fj.201802639R 30922079

[B56] Zhai J. Gao C. Fu L. Jing L. Dang S. Zheng S. (2018). Integrative Analyses of Transcriptome Sequencing Identify Functional miRNAs in the Chicken Embryo Fibroblasts Cells Infected with Reticuloendotheliosis Virus. Front. Genet. 9, 340. 10.3389/fgene.2018.00340 30233638 PMC6128223

[B57] Zhang J. Zhang X. Li Z. Wang Q. Shi Y. Jiang X. (2021). The miR-124-3p/Neuropilin-1 Axis Contributes to the Proliferation and Metastasis of Triple-Negative Breast Cancer Cells and Co-Activates the TGF-β Pathway. Front. Oncol. 11, 654672. 10.3389/fonc.2021.654672 33912463 PMC8072051

[B58] Zhang Z. Li J. Huang Y. Peng W. Qian W. Gu J. (2018). Upregulated miR-1258 Regulates Cell Cycle and Inhibits Cell Proliferation by Directly Targeting E2F8 in CRC. Cell Prolif. 51 (6), e12505. 10.1111/cpr.12505 30144184 PMC6528920

[B59] Zhao W. Steinfeld J. B. Liang F. Chen X. Maranon D. G. Jian Ma C. (2017). BRCA1-BARD1 Promotes RAD51-Mediated Homologous DNA Pairing. Nature 550 (7676), 360–365. 10.1038/nature24060 28976962 PMC5800781

[B60] Zhao Y. Yang J. Liu Y. Fan J. Yang H. (2019). HSV-2-Encoded miRNA-H4 Regulates Cell Cycle Progression and Act-D-Induced Apoptosis in HeLa Cells by Targeting CDKL2 and CDKN2A. Virol. Sin. 34 (3), 278–286. 10.1007/s12250-019-00101-8 30953292 PMC6599507

[B61] Zhao Y. Zhu C. Chang Q. Peng P. Yang J. Liu C. (2020). MiR-424-5p Regulates Cell Cycle and Inhibits Proliferation of Hepatocellular Carcinoma Cells by Targeting E2F7. PLoS One 15 (11), e0242179. 10.1371/journal.pone.0242179 33201900 PMC7671513

